# Counter-Irritation, Its Principles and Practice; Illustrated by One Hundred Cases of the Most Painful and Important Diseases Effectually Cured by External Applications

**Published:** 1838-10

**Authors:** 


### ART. XIV.

*Counter-Irritation, its Principles and Practice; illustrated by One Hundred Cases of the most painful and important Diseases effectually cured by External Applications.* By A. B. Granville, m.d., f.r.s., &c. &c. &c.—*London*, 1838. 8vo. pp. 353.

The task of noticing a work of this kind is the most disagreeable that can possibly devolve upon a reviewer. To pass it over entirely would be dishonest; to speak of it with approbation is impossible; and to speak of it as it deserves would be to employ severer language than we wish to use. If the first impressions conveyed by the general aspect of the work, and an inspection of its divisions and headings, were to be taken as truly answerable to its design and end, we should pronounce it an ingenious but unblushing advertisement of a secret remedy; and as such, out of the pale of respectable medical literature. If we hesitate to do so, it must be ascribed to the reluctance we feel to utter such a sentence on any production of the pen of a writer whose ability, education, experience, and station in society, all force upon us the admission that a purpose inconsistent with the principles commonly held to be honorable by the medical profession, must be the last he could entertain. At the same time we deplore the want of judgment betrayed by addressing such a work to the public, since we feel assured that the general opinion of the profession will be much less favorable to the intentions of the writer than that which we have professed ourselves anxious to entertain.

But it is especially necessary to remember, that this work is not addressed to the profession, and that it is solely meant for the public. If the single application of which it is designed to illustrate the extraordinary efficacy, is kept a secret from the first page to the last, we are disabled from saying that the author has been actuated by any other than a prudent desire to withhold from the unprofessional the ready mischief of a formula. No doubt it might have been better to address a few words to the profession alone, and to mention the combination of ingredients asserted to be so remarkably useful; but we have no right to expect, in any work, incompatible excellencies. A book designed to enlarge the empire of counter-irritation, by placing in the hands of all practitioners a means found more serviceable than any previously known formula of external application, would be received with gratitude as an important contribution to therapeutics. The truth it contained, although very valuable, might also be conveyed in a few words. But such is not Dr. Granville's intention. We have no right, therefore, we repeat, to say to him, your book might have been compressed into a few pages, and the exact composition of your almost infallible liniment should have been communicated. On the contrary, we are compelled to say, you have become deeply impressed with a sense of the value of counter-irritation; and by years of reflection and study have been, under Providence, instrumental in discovering a combination which had escaped previous investigation; had not been accorded to the physicians of the East, nor to the more scientific labours of Western Europe; had eluded the perspicacity of Le Fay, St. John Long, and Dr. Turnbull. You have found this application in many instances of instantaneous service, and in none without eventual benefit. You are profoundly convinced that its application requires great physiological discernment; and to be applied safely, must be applied by your own hand. You are seriously impressed with the objections entertained by the public to external modes of treatment, and see, with regret, an enormous and useless expenditure of drugs. All this you solemnly say; and you write, therefore, to the public, to show them, by examples, how superior is this new mode of external treatment, how agreeable, how efficacious; and yet you refrain from enabling them, by the use of the specific liniment you have discovered, to do themselves mischief by having indiscriminate recourse to it. The only business of a reviewer, therefore, is to see how this undertaking has been performed.

The conclusions to which Dr. Granville has arrived are thus conveniently stated in his concluding pages.

"1st. That there exists a species of external treatment, by which a great many very important disorders of the human frame hitherto considered as incurable, or difficult of cure, may be speedily and successfully cured without having recourse to internal remedies.

"2d. That although, from time immemorial, several agents of known power have been and are still employed in the external treatment of diseases; nevertheless, the various ammoniated spirituous preparations described and recommended in the present volume, have never before been offered to the public, although they possess much greater energy for carrying on and expediting that treatment.

"3d. That even where the diseases are of a nature to require the use of internal remedies, the same ammoniated spirituous preparations, externally used, will be found to be a most powerful auxiliary in hastening and securing the good effect of those remedies.

"4th. That in several instances, by the rapid and almost instantaneous manner in which they act, the ammoniated spirituous preparations have been the means of saving life from imminent danger.

"5th. That the principle on which all such external agents are supposed to act in the cure or alleviation of human maladies, has been termed COUNTER-IRRITATION; but that, in adopting such term, many of the phenomena which accompany the use of ammoniated external applications are left unexplained.

"6th and lastly. That by promoting a more general adoption of a counter-irritating or external treatment of disease, and thereby saving the constitution of patients from the pernicious effect of a polypharmacous treatment, a great service would be rendered to the public, and an important era established in the annals of practical medicine." (p. 352.)

Here we see, very perspicuously set forth, the value of counter-irritation in general; the superior value of Dr. Granville's secret preparation; its rapid action; its almost universal utility. The title-page (to turn from the last page to the first) declares that these views are illustrated by "one hundred cases effectually cured." The preface confirms the hopes of the reader, and contains what, in the peculiar circumstances of the case, is a piece of indispensable information, the exact address of the author, the number of his house, the name of his street, and that of the nearest fashionable square.

If we might presume to say a word of the first four sections out of the nine into which this work is divided, we should say that they were rather above the comprehension of the general reader: but persons more experienced than ourselves in the state of the public mind in London, consider this as a very trifling objection in medical writings. We should have considered Section V. as a better commencement, in which the field is pretty well cleared of all former counter-irritations. Not that Dr. Granville speaks illiberally of Le Fay's pomatum, "supposed to consist principally of hellebore," or of "the practice of the late Mr. St. John Long," who is "believed to have employed the milky juices of Euphorbiaceous plants, diluted with gummy mucilages;" or of the "remedies eulogized by Dr. Turnbull." But he sweeps all their applications off the stage, as being, although sometimes productive of good effects, dangerous, "because their poisonous principles may be absorbed into the system." In the cases of death occasioned by St. John Long's secret embrocation, of which the facts came to our particular knowledge, the fatal event was not attributable to this cause. We hope Dr. Granville's secret embrocation is less dangerous: no doubt it would be more scientifically applied. St. John Long derived his knowledge from his studies as a coach-painter; Dr. Granville's education has been of a different kind. They only resemble in keeping their remedies secret. Poor St. John Long probably took his secret out of the world with him; and will, we trust, find it useful in his own defence when confronted with his victims. Dr. Granville, we are assured, will impart his to the world, or at least to some well selected successor before he dies. The communication could only properly be made to one whose circumstances would place him above suspicion.

There is something ingenious in the headings of the sections of the work before us, which we cannot overlook. Section sixth, for instance, treats "of Ammoniated Counter-Irritants, *and particularly of Anti-dynous Lotions, a species of powerful External Applications,* capable of producing all the Phenomena of ordinary artificial Counter-Irritation, *and Something more, which the usual Counter-Irritants have hitherto failed* to produce." In this section Dr. Granville disposes of the merits of the actual cautery, boiling water, mineral acids, the usual preparations of ammonia, and at least one hundred and three preparations of cantharides, by this single observation: "that as it is impossible to fix or determine the precise dose of the powerful agent contained in them, which will enter or affect the animal system when used externally, so we cannot predicate the quantity of effect it will produce beyond what may be desired—particularly on such parts of the constitution as ought not to be affected at all, and which cannot be affected by the said agent without material injury. The utmost caution, therefore, is necessary in the use of all such counter-irritants; whereas *the ammoniated preparations I recommend* are neither obnoxious to such objections, nor do they require any such caution." Dr. G. is not answerable for the *italics;* but we conceive they do not detract from the force, or even from the meaning of the passage quoted. The truth to which they give, we venture to say, more prominence, is evidently that which Dr. Granville is very anxious to convey, as in the same page he intimates that his secret preparation produces a "peculiar and wished-for effect," without any of the drawbacks attendant upon all previously known authorities.

To this useful counter-irritant, Dr. Granville gave the name of Antidynous, simply from its instantaneous effect in relieving pain, which he says, very unnecessarily, "is a positive fact." The removal of pain is not always, he confesses, permanent; but then, on the other hand, the

*Antidynous* application is capable of producing other striking effects. In "TIC, dependent on *organic* mischief done to the nerves or brain," the pain is, "at every attack" suspended; but "not so in Tic-douloureux, which is the consequence of sympathetic action between certain organs," for then "the relief produced by these preparations" (*the antidynous*) "is permanent." (p. 52.) This relief is almost "magically rapid." A very excellent blister is raised at the same time. Dr. Granville thinks it acts on "the nervous papillary terminations which it deadens;" this effect being continued or projected to the other extremity" (of the nerve) "in which lies the morbid pain to be relieved." (p. 54.) The case of the Countess of ——— is mentioned to illustrate this theory: she was relieved of spasms in the back in thirty-five seconds: "all pain ceased, not only in the parts nearest to the application, but also in the most remote parts of the spinal column." Of course the application was of "suitable strength;" yet it neither produced heat, "much less rubefaction," in the time mentioned. The effect might possibly be produced, Dr. G. admits, by "absorption of the volatile particles of the ingredient used," or their transmission along the sheath of the implicated nerve, after the manner of the electric fluid. But the fact is certain, "nor can there," he says, "be the smallest fear of contradiction to the assertor of it," that the antidynous lotion is a most important addition to the class of counter-irritants. Indeed, until medical men are acquainted with its composition, contradiction is plainly out of the question. But the public are not encouraged to any rashness; for Dr. G. thus impressively concludes section vi.

"I may add, likewise, that in not a single instance of their application *under my immediate notice* have I witnessed the smallest injury done to the part; although, on a very few occasions, either through design or neglect, and not unfrequently from an inopportune and injudicious use of them *by persons unacquainted with their real power*, extensive vesication, and an abraded ulcerated surface have been produced, where no such results were required or desirable." (p. 56.)

Again we plead guilty of the *italics;* but, simply observing that no one but the inventor *can* well be acquainted with the real power of a secret medicine, we think the public can scarcely be so stupid as not to understand these warnings.

The seventh section of Dr. Granville's work is partly devoted to showing the use of natural counter-irritation, in the shape of habitual ulcers, eruptions, &c., on the danger of curing which we are disposed to think he lays an undue stress; and partly, to repeating that the antidynous lotion is the best, the safest, and the most generally successful of all external applications; and that under its use *long attendances* are completely put an end to. It cures immediately, and safely, and not unpleasantly. The fashionable physicians of Rome, skilled in all the attractive arts and imbued with the science of Greece, promised, we read, no less to the aristocratic circles of the imperial city two thousand years ago: but to this day the word of promise has been kept only to the ear. The ammoniated lotion may fulfil it to the sense. For not polypharmacy, not veratria-rubbings, not infinitesimal doses, nor any nor all other means or appliances possess all the advantages of the antidynous lotions. If Dr. Turnbull, or Dr. Quin, or indeed any other fashionable practitioners, continue to be the oracles of the aristocracy, no blame can henceforth be attributed to Dr. Granville. His candour as to the effects of his lotion is equal to his reserve as to its nature. Its composition is indeed unknown; but he does not conceal the fact that it is able to render all ordinary medical attendance unnecessary. Apothecaries may abscond, and doctors depart, and druggists drown. The painful cures which they effect in weeks, of which the records are too often read in voluminous diaries of expense, are wrought by the antidynous lotion in a given number of minutes, often without any physic given, and generally with very few fees received. It will be in vain that the profession resist this scheme: the public must adopt it with one voice. If any one doubts this, or suspects us of over-rating Dr. Granville's pretensions, let him carefully read the conclusion of the eighth section of his work.

We fear that many medical readers, however, not being acquainted with Dr. Granville's predetermination to omit the formula for an application the subject of so much promise and so much praise, may be led on with a delusive hope from chapter to chapter, only to be disappointed in the end. Dr. Granville dies, and makes no sign. Every now and then the secret seems about to be revealed, yet it is never divulged. The whole book is a long riddle, an elaborate enigma. We are acquainted with a country doctor who cures all sorts of dropsy by a secret medicine. Nothing can be more candid than our friend: we see him many times in a year, and he communicates his success as unreservedly as Dr. Granville; but he tantalizes us no less by his very communicativeness. Again and again he tells us that his medicine is perfectly safe, and very pleasant, and always successful; and ever does he wind up by saying that it consists merely of—"a few simples." Dr. Granville's book has reminded us of our country friend an hundred times. The antidynous lotion is ever held up almost in view; it is said to rubefaciate, to vesicate, and to relieve pain; it is called indifferently antidynous or ammoniated: everything is done except to declare its composition. Just when the reader's curiosity begins to flag, section nine is given, the last in the book, not to reveal the great secret, but to provoke curiosity the more. In this section the lotion is described by its properties, but still left, after the enigmatical model, undefined; as if merely to exercise the reader's wits.

As to the choice of *place* of application of the lotion, which some may consider an easy matter, Dr. Granville tells us it "must depend on our own intimate knowledge of the relation that exists between certain inward organs and certain portions of the skin." (p. 78.) As to the choice of *time*, the ingenious reader may perhaps surmise the truth, that the sooner the "antidynous or ammoniated lotions" are applied the better. Nervous headachs, which these lotions always cure, are cited in illustration of this: nevertheless, in this, and in all other cases, "a minute examination of the case itself" is required. We are glad that Dr. G. has inserted this caution; for the druggists are sure, before long, to advertise "Dr. Granville's Lotion." The *duration* of the application is seldom to be longer than from one to six or eight minutes. Less than one minute is often sufficient; but ten or twelve minutes are necessary for vesication and cauterization. Sometimes Dr. G. has deemed it necessary to bind the external application to the part, and leave it on till dry; "a result which takes place very speedily, owing to the almost ethereal volatility of the lotions." (p. 83.) In this page, in addition to this characteristic, we learn that the lotion is "colourless and transparent." The *mode of application* is by impregnating with it a piece of linen folded six or seven times, or a piece of thick coarse flannel. One or the other of these being placed on the spot chosen, is to be pressed very steadily and firmly with a thick towel doubled several times, or with an instrument, also invented by Dr. Granville for this particular purpose.

One-fifth of the volume being devoted to the account of the undescribed lotions, four-fifths are filled up with a narrative of cases; comprising every variety of suffering, from epilepsy and trismus to a severe blow on the nose; besides a "fall from a lofty haystack." These cases Dr. G. pronounces to be "interesting and attractive;" and they are presented as mere selections from nine years' practice.

It is painful to us to observe a physician of Dr. Granville's standing labouring under an evident fear that his simple assertions will not be believed; and it is to this unreasonable suspicion that we are indebted, it appears, (p. 97,) for several particulars in the record of the cases which, to say the least of them, add nothing to their authenticity. We, at least, have no desire to doubt the truth of every word in each of the cases; and we, therefore, greatly lament the manner in which they are detailed. The titles of the patients and their letters might, Dr. Granville cannot but know, be far overmatched by the learned Dr. Morison; and the *facts* they relate are often exactly such as the writers are least fitted to be the historians of. A gentleman of Dr. Granville's experience must be well aware that, in the case of the veriest quack medicine, nobody doubts the relief given: all the question is about the disease. We regret, therefore, to see that Dr. Granville's patients write to "meet the wish" of the doctor: we are suspicious when they beg to remind him that "he will remember" so and so: in short, when they write like people *desired* to write a letter that can be published. The newspapers, those great organs of the dissemination of truth, abound with letters quite as well done. Such patients always have somebody to thank for the inestimable blessing of Doctor Somebody's attendance; and, under Providence, they are always obtaining benefit, after trying all the rest of the faculty in vain.

Case iv. is that of "the Right Hon. the Countess of ——." "Her ladyship" had a pain in her chin. The lotion was applied with great accuracy to "that part of the chin which corresponds with the opening through which the mandibulo-labialis branch of the fifth nerve comes out." Common readers, for whose sake Dr. G. expressly says that his book is written, "free from technicalities and scholastic definitions," will be doubtless impressed with considerable awe on finding their simple chins divisible into so many parts. The right honorable patient was cured for a whole week: then the pain recurred, or was inclined to recur, and prevented by the application of *one* leech. Then we have one case of "a young lady of Southampton, remarkable for her general appearance of perfect health and personal attractions, lately married;" Lady Caroline ——, a young and unmarried daughter of the Earl of ——; and the young son of Lord F— L— G—, with an F— L— G— letter, full of proper acknowledgments. We have "noblemen," and "gentlemen filling considerable situations under government;" another countess "closely allied to two of the first families in this country;" and the "Duchess of ——" in her chamber; we have "a noble earl, not more conspicuous for his station in society, which he has, through a long career, enhanced by great public and private worth, than proverbially known for being at one time the victim of the severest form of nervous headach;" we have ladies of "wealthy baronets;" and soul-thrilling notes from persons of high rank, of which the purport is, "Pray do come this evening, without fail, to Stanhope street, if you cannot dine with us; for the Countess —— is suffering dreadfully from faceach," (p. 225;) and moving incidents on the occasion of Dr. G. going out of town to dine "with a nobleman," and finding "Viscountess —— in the drawing-room, dressed for dinner," with a very severe toothach; when "fortunately there was in the house a bottle of ammoniated counter-irritating lotion;" and Lord T——, when he came home to dinner, as he "occupied a ministerial station of high importance," heard the whole story of toothach and wonderful cure at once. We were wondering, when reading the beginning of this case, where the lotion was to come from; and it advantageously illustrates the propriety of keeping a bottle ready. But where is the lotion made; where to be bought? Surely it cannot be a fact—a *positive* fact, as Dr. G. says of one of his own,—that it can only be procured on application at 16, Grafton street, Berkeley square! It is impossible to believe this. Is not Dr. Granville a member of the British Medical Association, a body so jealous of physicians, and so irate against all irregular proceedings? With what face, with what decency, could bodies of men and learned associations petition for the annihilation of unfortunate quacks, if such proceedings were thought venial in regular physicians, Fellows of the Royal Society, and whose practice is amongst the nobility and gentry of the land? This would be, as with Sunday legislation, where the humble alone are stricken, and the elevated offender is secure; as poor rogues hang whilst richer ones only go abroad.

Of Dr. Granville's talents and knowledge there can be but one opinion. It is therefore as painful as it is astonishing to see him quit the sober and legitimate path which he has hitherto trod, and hazard the dreadful chance of being confounded with men every way his inferiors. That he does not do this from mere obtuseness of feeling, nor from ignorance, is too clearly revealed by the pains he has taken (by direct communications to the editors) to conciliate the favour of the medical periodical press to his *omissions*,—to call them by the gentle name which he himself employs to designate them. In these communications he professes not to consider it incumbent upon him to reveal to the profession what he regards as a valuable secret obtained by his own industry and ingenuity; and that having, in his opinion, told them, clearly and without mystification, that his lotion consists of a solution of ammonia, stronger than any in the London Pharmacopœia, he disclaims all idea of injustice when he leaves them where he himself began. He does not think it necessary to mention the exact strength of the lotion employed in any case; and he professes to have explained that the vehicle of solution is any spirituous distillation of aromatic herbs and camphor; the strength to be apportioned to the particular case. Having discovered, by experiments *on himself*, what strength of lotion to employ, he leaves others to do the same. In truth, Dr. Granville is far from being clear and explicit even to this extent; but speaks of his lotions as formed by regulating the proportions of "ammonia, spirits of wine, vegeto-aromatic spirits, camphor, and other stimulating and evaporable substances, differing from the few preparations already in use, and combined with water, or with oils, or butyraceous vehicles, or saponified into cerates." (p. 51.) We can scarcely imagine anything less lucid than this. Giving Dr. Granville all possible advantage of his plea, what is the deduction to which we are driven? that his book is not written to give the profession any information at all; but merely to tell the public that he possesses a means of giving relief, which he refrains, for his private emolument, from adding to the resources of the medical art. We do not believe that Dr. Granville approves of this kind of ethics, and we lament to see him affect to adopt it.

---

